# Antidepressant effects of a Persian herbal formula on mice with chronic unpredictable mild stress

**DOI:** 10.22038/AJP.2023.22191

**Published:** 2023

**Authors:** Hakimeh Gavzan, Atefeh Araghi, Behrokh Marzban Abbasabadi, Nastaran Talebpour, Hannaneh Golshahi

**Affiliations:** 1 *Department of Basic Sciences, Faculty of Veterinary Medicine, Amol University of Special Modern Technologies, Amol, Iran*; 2 *Department of Clinical Sciences, Faculty of Veterinary Medicine, Amol University of Special Modern Technologies, Amol, Iran*; 3 *Nanobiotechnology Research Center, Avicenna Research Institute, ACERCR, Tehran, Iran*

**Keywords:** Anxiety, Biochemical parameters, CUMS, Depression, Stress

## Abstract

**Objective:**

Depression is a serious mental disorder. Despite numerous medications, there are still limitations in depression treatment. So, herbal medicine has been considered an alternative therapy. This survey evaluated the effects of a Persian herbal formula on mice with chronic unpredictable mild stress (CUMS).

**Materials and Methods:**

A combination of *Aloysia triphylla* *citrodora,* *Citrus aurantium*, *Echium amoneum*, *Lavandula angustifolia*, *Melissa officinalis*, *Salix aegyptiaca*, *Valeriana officinalis*, *Viola odorata*, and *Cinnamomum zeylanicum* was prepared. Except for the control group, animals were subjected to CUMS for 8 weeks in 5 groups (n=10): CUMS, vehicle (distilled water), herbal formula (0.23 ml/mouse), fluoxetine (20 mg/kg), and bupropion (15 mg/kg). All administrations were performed orally daily for the last 4 weeks. The depression and anxiety-like behaviors were assessed by sucrose preference (SPT), tail suspension (TST), forced swimming (FST), and elevated plus-maze (EPM) tests. Superoxidase-dismutase (SOD) activities in tissues, and serum levels of cortisol, alanine-aminotransferase (ALT), and creatinine were measured. Also, histopathological changes were evaluated.

**Results:**

This formula significantly increased SPT (p<0.001) and decreased immobility time in FST and TST (p<0.01), but it was not effective on EPM vs. CUMS mice. The herbal formula did not change the serum level of creatinine or ALT, but insignificantly reduced cortisol vs. CUMS and vehicle groups. SOD activity increased in the brain vs. vehicle group (p<0.05). There were no changes in histological examination.

**Conclusion:**

The herbal formula improved depression-like behaviors which are possibly related to its anti-oxidative effect on the brain. Also, it did not cause any negative changes in the biochemical and histopathological analysis.

## Introduction

Depression, which is characterized by sorrow and low mood, is one of the most frequent psychological disorders. In spite of the availability of a variety of antidepressants, several obstacles need to be overcome to treat depression. Inefficacy, side effects, the delayed onset of action for some antidepressants, and treatment resistance ( Rafeyan et al., 2020 ▶) necessitate searching for safer and more efficient antidepressants.

Herbal remedies are usually recommended as an alternative treatment for psychiatric disorders. Medicinal plants have multiple bioactive compounds which can intensify each other’s therapeutic effects and relieve their side effects. Also, the combination of these components may positively change the metabolism and absorption of each other (Williamson, 2001 ▶). Besides, administration of a herbal drug with multi-effective components or a mixture of several herbal remedies could be effective against multi targets of complex diseases such as depression and anxiety (Sarris et al., 2011 ▶). For long centuries, Persian herbal remedies have been used to cure mental disorders such as depression (Jalali et al., 2021 ▶). Poly-herbal therapy is a common strategy used in traditional medicine (Che et al., 2013 ▶). Iranians use a new poly-herbal formulation to treat or prevent different psychological disorders such as depression and anxiety. The formulation comprises nine Persian herbal drugs: *Aloysia citrodora, Citrus aurantium, Echium amoneum, Lavandula angustifolia, Melissa officinalis, Salix aegyptiaca, Valeriana officinalis, Viola odorata, and Cinnamomum zeylanicum.* However, there has not been any research on the efficacy and action mechanism of the combination against depression. 

Based on experimental and clinical studies, these herbal drugs alone showed antidepressant and anxiolytic effects (Akhondzadeh et al., 2003 ▶; Aryanezhad et al., 2021 ▶; Becker et al., 2014 ▶; Ghazizadeh et al., 2020 ▶; Hattesohl et al., 2008 ▶; Karim et al., 2018 ▶; Komaki et al., 2015 ▶; Sayyah et al., 2006 ▶; Upadhyay et al., 2016 ▶). Furthermore, these herbal drugs contain several bioactive components including flavonoids, caffeic acid and phenylpropanoids which are effective against neurological disorders such as depression and anxiety. Anthocyanins, tannin, and luteolin as the flavonoid and cinnamaldehyde as the phenylpropanoid, and verbascoside and rosmaric acid are the important constituents in these plants. that showed the antidepressant effects (Chandrasekhar et al., 2017 ▶; Takeda et al., 2002 ▶; Zhu et al., 2019 ▶). 

Depression induced by stress leads to complex neurological alterations. There are many studies which demonstrate the relation between the neuropathology of major depression and the dysregulation of hypothalamic-pituitary-adrenal (HPA) axis (Juruena et al., 2018 ▶; Bai et al., 2018 ▶). Due to the hyperactivation of HPA axis, the concentration of cortisol increases in the serum of patient with major depression (Keller et al., 2017 ▶). On the other hand, it has been reported that the antidepressant agents reverse the increased level of circulating glucocorticoid (Himmerich et al., 2007 ▶). Oxidative stress is another pathway which plays a role in the neurobiology of depression (Vaváková et al., 2015 ▶). Therefore, attenuation of the oxidative stress and enhancing the anti-oxidative defense system has been considered for treatment of depression. 

**Figure 1:**
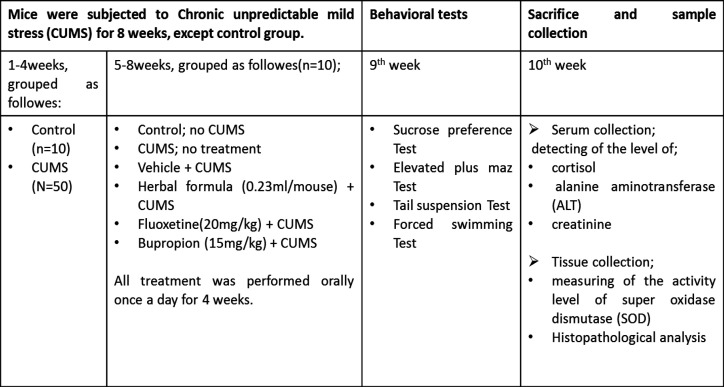
The schedule of the experimental design

Due to the importance of evaluating the effects of herbal formulation in animal experiments, for the first time, we assessed the effect of the sub-chronic administration of the Persian herbal formula in a mice model of chronic unpredictable mild stress (CUMS). CUMS is a well-established animal model of depression and imitates many symptoms of human depression-like anhedonia (Antoniuk et al., 2019 ▶). It is demonstrated that CUMS procedures alter the HPA axis activity (Wei et al., 2017 ▶) and oxidative stress in animals as well as depressive patients (Che et al., 2015 ▶). First, depression- and anxiety-like behaviors were tested by sucrose preference test (SPT), tail suspension test (TST), forced swimming test (FST) and elevated plus maze (EPM). Additionally, the level of cortisol in serum and superoxide dismutase (SOD) activity in the brain, kidney, and liver were measured in order to evaluate the possible regulatory action of this Persian herbal formula on the HPA axis and anti-oxidative defense system. Since administration of some components of herbal drugs may have adverse effects on the body organs, the liver and kidney functional markers and histopathological analyses were carried out at the end of the treatment (Figure 1). 

## Materials and Methods

**Animal**

Adult male NMRI mice (20–25 g, Pasteur Institute of Iran-Amol) were housed in the standard situation (23 ± 2.0°C and 12 h light/dark cycle (6:00–18:00)) and fed *ad libitum* with rodent’s chow and water. All experiments were performed based on the protocols set by Research Ethics Committee of Amol University of Special Modern Technologies (Specific code: IR.ASMT.REC.1401.003).

**CUMS procedure**

CUMS was implemented according to  a methods (Mao et al., 2009 ▶). The mice experienced several mild stressors on a weekly schedule for 4 weeks. These included: 1 and 2) deprivation of food and water for 24 hr, respectively, 3) exposure to an empty bottle for 1 hr, 4) experiencing 24 hr of reversed light/dark, 5) 6 hr cage tilting by 45°, 6) being kept in the wet cage for 24 hr (with water 200 ml/100 sawdust bedding), 7) physical restraint for 2 hr, and 8) exposure to a foreign object for 24 hr.

**Drugs and administration**

The combination of *Aloysia citrodora, Citrus aurantium, Echium amoneum, Lavandula angustifolia, Melissa officinalis, Salix aegyptiaca, Valeriana officinalis, Viola odorata, and Cinnamomum zeylanicum,* the herbal formula was provided by Parsiteb Company (health certification code: 94505: A61K00/36. (Fluoxetine) and Bupropion (Abidi Company, Iran) were dissolved in distilled water. The experimental group (n=10) included: 1) control (no-CUMS); 2) CUMS (no administration); 3) Vehicle (distilled water); 4) the herbal formula (0.23 ml/mouse), 5) Fluoxetine (20 mg/kg), and 6) Bupropion (15 mg/kg). The administration was done daily for four weeks by intra-gastric gavage (10 ml/kg). During the treatment, CUMS progress was completely observed. The dosages of fluoxetine and bupropion were selected from the last studies (Dhir and Kulkarni, 2007 ▶; Yi et al., 2003 ▶). The volume of administration of the herbal formula was chosen based on the human and mouse dose conversion formula (Liu et al., 2019 ▶).

**Behavioral test**

**Sucrose preference test (SPT)**

At the end of the treatment, SPT was carried out as previously explained with minor modifications (Mao et al., 2009 ▶) to assess the reaction of depressed animals to positive emotional stimuli and anhedonia. SPT was performed through 5 days; on the first day, we put two bottles of sucrose solution (1% w/v) in each cage of the mice. On the second day, one of the bottles was switched with water. On the first and second days, mice were trained to consume the sucrose solution. On the third day, the mice were deprived of water and food. Then SPT was conducted on the fourth and fifth days. On the fourth day, we put every single mouse in a separate cage with food and 1% sucrose solution, and fresh water bottles. On the fifth day, both bottles were weighed to measure the consumption of sucrose and water.

We used the following formula to calculate the sucrose preference%: 

sucrose consumption/ (sucrose consumption+ water consumption) *100

**Elevated plus-maze (EPM) test**

EPM test was performed according to a described procedure (Yen, et al., 2020 ▶). EPM had two closed and two open arms (30 cm long, 5 cm wide, and 15 cm height of the closed arms, 5*5 cm central square platform, 80 cm height from the floor). The ratio of the time in open to closed arms was measured through the following formula: 

the time spent in open arm / the total time spent in the open and closed arms

**The tail suspension tests (TST) and forced swimming test (FST)**

TST and FST were done based on previously reported methods (Mao et al., 2009 ▶; Yan et al., 2020 ▶). In TST, the mice were suspended from 50 cm height by their tail. In FST, the animal was put into a glass cylinder (10 cm diameter and 25 cm height) containing 15 cm of water (25±1°C). After 1 min of adaptation, the immobility time was recorded for 5 min.

**Sample collection**

At the end of the procedure, the animal was anesthetized with ether to take blood samples from the cardiac puncture. Then, samples were divided into two parts. For biochemical analysis, one part of samples was centrifuged (5000 rpm for 15 min) to get serum. For hematological analysis, another part of samples was put into K2EDTA Tubs (BD Microtainer, USA). The kidney, liver, intestine, and brain were dissected and part of which was transferred into a liquid nitrogen tank and kept at -80°C to determine superoxide dismutase (SOD) activity. The remaining tissues were put in 10% natural buffered saline for histological analysis.

**Biochemical analysis**

The serum content of Alanine aminotransferase (ALT) and creatinine was detected by the serum chemistry kit (HITACHI 7180, Japan). Moreover, the serum level of cortisol was measured by the Elisa kit of the Bioassay System (Bioassay System, CA, USA Koma Biotech, CAS#NUMBER K0331230P, Soul, Korea). The activity level of SOD was estimated by the corresponding commercial assay kits (zellBioi GmbH, Germany) according to the manufacturer’s instructions using a spectrophotometric method.

**Statistical analysis**

The statistical analysis was performed by the SPSS software version 22.0 for windows. The normality of data was checked by Shapiro-Wilk test. All data are expressed as mean±SEM and the normally distributed data was analyzed by one-way analysis of variance with Turkey’s post hoc test. A p<0.05 was considered significant. The graphs were drawn by the GraphPad Prism software version 8.

## Results

**Effects of the Persian herbal formula on depression-like behaviors in the CUMS model**

**Figure 2:**
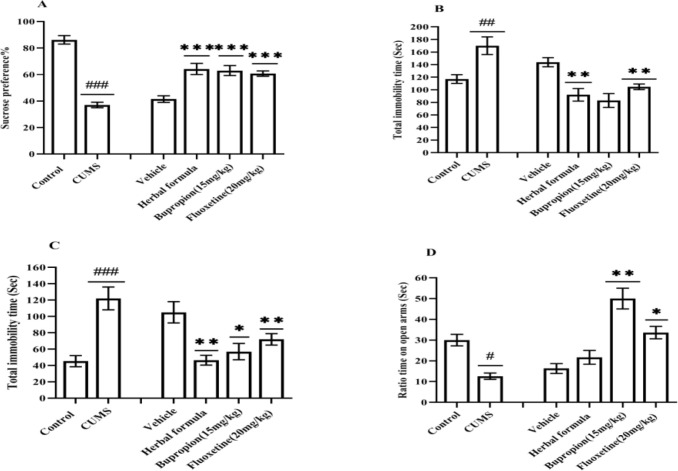
Effects of the herbal formula on A) sucrose preference test B) tail suspension test, C) forced swim test, and D) elevated plus-maze test. The data represent mean±SEM. CUMS; Chronic unpredictable mild stress. Fluoxetine20; Fluoxetine (20 mg/kg). Bupropion15; Bupropion (15 mg/kg). #, ## and ###p<0.05, <0.01, and <0.001 respectively vs. the control. *, ** and ***p<0.05, <0.01, <0.001 respectively vs. the Vehicle

As shown in Figure 1A, the sucrose preference% was significantly decreased in the CUMS group compared to the control group (p<<0.001). Herbal formula as well as antidepressant drugs raised the sucrose preference% in comparison to the vehicle (p<<0.001) (Figure 2A). The immobility time significantly increased in the CUMS vs. the control group in both TST and FST (p<<0.01 and 0.001, respectively). In TST, the herbal formula as well as antidepressant drugs decreased the immobility time compared to that of the vehicle. This reduction was significant in herbal formula and bupropion group (p<<0.01) (Figure 2B). In FST, the herbal formula and fluoxetine resulted in a decrease in the immobility time vs. that of the vehicle (p<<0.01, 0.05, and 0.01, respectively) (Figure 2C).

**Effects of the Persian herbal formula on anxiety-like behaviors in the CUMS model**

The time ratio of open arms significantly reduced in the CUMS mice vs. the control (p<<0.05). Fluoxetine and bupropion increased the time ratio of open arms vs. the vehicle (p<<0.05 and 0.01, respectively). Although the herbal formula could increase this ratio compared to the vehicle, its effect was not significant (Figure 2D). 

**Effects of the Persian herbal formula on the liver and kidney functional parameters of the CUMS animal**

**Figure 3:**
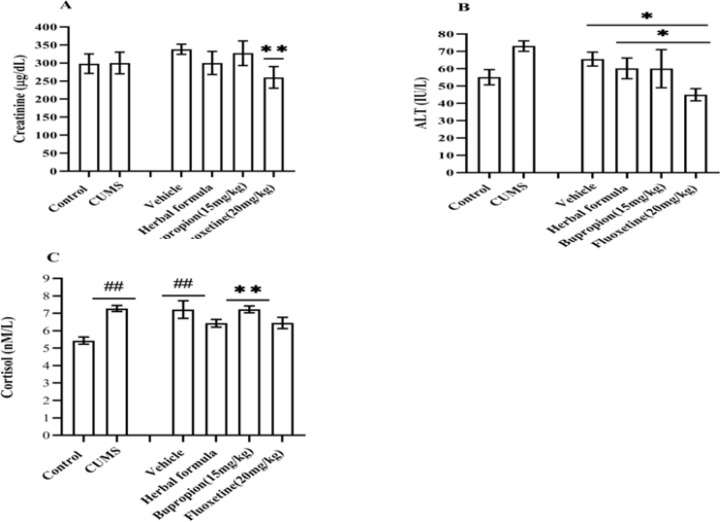
Effects of herbal formula on the serum level of A) Creatinine, B) ALT and C) Cortisol. The data represent mean±SEM. CUMS; Chronic unpredictable mild stress. Fluoxetine20; Fluoxetine (20 mg/kg). Bupropion15; Bupropion (15 mg/kg). ##p<<0.01 vs Control, *p<<0.05 vs. the vehicle and herbal formula, **p<<0.01 vs. the vehicle

The level of creatinine decreased in the herbal formula and fluoxetine vs. the vehicle group (p<0.01), but this change was only significant in the fluoxetine group (Figure 3A). As shown in Figure 2B, the ALT level insignificantly increased in the CUMS group in comparison to all other treatment groups. Also, a significant decrease of ALT level was witnessed in the fluoxetine group vs. the vehicle, herbal formula (p<0.05), and CUMS groups (p<0.01) (Figure 3B).

**Effects of the Persian herbal formula on the cortisol level in the serum of CUMS animal**

The cortisol level significantly increased in the CUMS, vehicle, and bupropion (p<0.01) vs. the control group (Figure 3C). Also, the herbal formula and fluoxetine increased the cortisol level, but their effect was not significant in comparison to the control and vehicle. 

**Effects of the Persian herbal formula on the SOD activity level in tissues of the CUMS animal**

**Figure 4:**
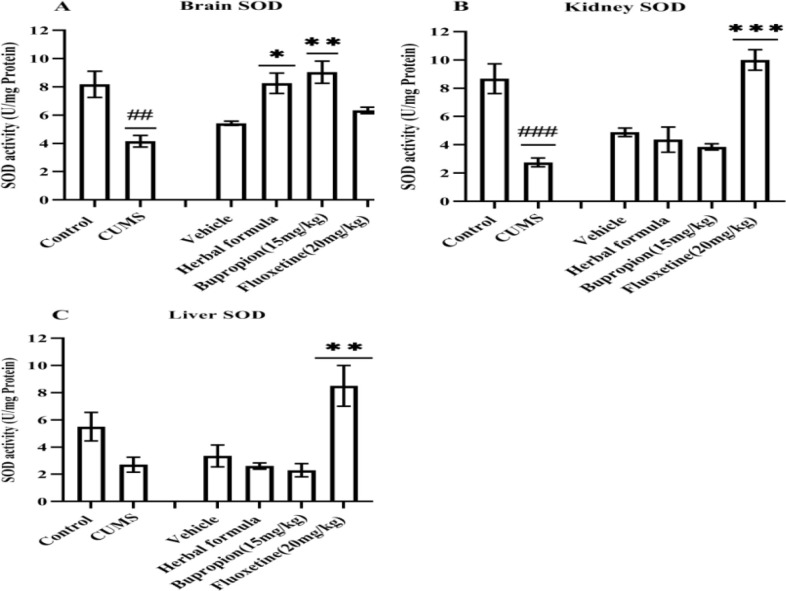
Effects of herbal formula on superoxide dismutase (SOD) activity of the A) Brain, B) Kidney, and C) Liver. The data represented the values of mean±SEM. CUMS; Chronic unpredictable mild stress. Fluoxetine20; Fluoxetine (20 mg/kg). Bupropion15; Bupropion (15 mg/kg). ## and ###p<0.01 and <0.001 respectively vs the control. *, ** and ***p<0.05, <0.01, and <0.001 respectively vs the vehicle

CUMS mice had a significant reduction in SOD activity of the brain and kidney (p<0.01 and <0.001, respectively) compared to the control (Figure 3A, B and C). In comparison to the vehicle, the herbal formula and bupropion raised the SOD activity in the brain (p<0.05 and p<0.01, respectively) and fluoxetine increased this activity in the liver and kidney (p<0.01 and 0.001, respectively) (Figure 4). Compared to the vehicle and control, the herbal formula was not able to increase the SOD activity in the kidney or liver. 

**Effects of the Persian herbal formula on the histopathological changes in the CUMS animal**

**Figure 5:**
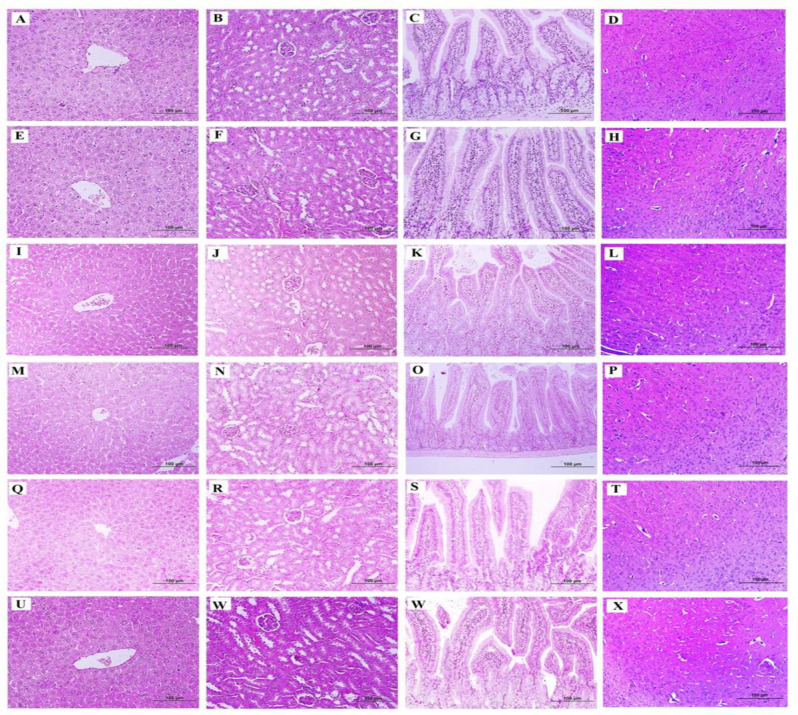
The histological evaluation of the herbal formula. H&E stained section of the liver, kidney, intestine, and brain, (A-D): control group, (E- H): Vehicle, (I- L): Fluoxetine 20, (M-P): Bupropion 15, (Q-T): CUMS, (U- X): herbal formula (H & E, × 200)

Generally, there were no remarkable lesions in the architecture of various organs in comparison with the control group (Figure 5).

## Discussion

For the first time, this study reports that the Sub-chronic consumption of the examined herbal formula for a month improved CUMS-induced depression-like behaviors while had no anxiolytic effect. 

The present results corroborate the previous studies reporting the antidepressant effect of some herbal ingredients of the present herbal formula. The oral consumption of Cinnamomum in a chronic manner induced antidepressant effects (Upadhyay et al, 2016 ▶). The hydroalcoholic extract of *Cinnamomum zeylanicum* for four weeks in CUMS mice had an increasing effect on sucrose preference and a decreasing effect on the immobility time in FST (Aryanezhad et al., 2021 ▶). Antidepressant and anxiolytic effects were observed after the administration of hydroalcoholic extract of *Melissa officinalis* for 14 days against the stress restrain model in mice (Ghazizadeh et al., 2020 ▶). The daily consumption of *Echium amoenum* aqueous extract for 6 weeks resulted in antidepressant effects in the major depressive patient (Sayyah et al., 2006 ▶). Antidepressant and anxiolytic effects were obtained after sub-acute oral treatment of methanolic and ethanolic extract of *Valeriana officinalis* in rats (Hattesohl et al 2008 ▶). Besides, there are some polyphenols in herbal formula with antidepressant effects. Luteolin, in a flavonoid of *Lavandula* *angustifolia*, had a decreasing effect on the immobility time in FST in ovariectomized rats (Zhu et al., 2019 ▶). Further, intraperitoneally administration of rosmarinic and caffeic acid, the major compounds of *M. officinalis*, inhibited the depressant-like behaviors in FST and TST in mice (Takeda et al., 2002 ▶). Cinnamaldehyde, the main constituent of Cinnamomum, inhibited the depressive-like behaviors in CUMS-rats (wang et al., 2020 ▶). Hesperidin, a flavonoid in *Aloysia citrodora*, had antidepressive effects on TST and FST in mice (souza et al., 2013 ▶). The i.p. administration of the flavonoids of *Viola odorata* exerted antidepressant-like effects on FST and TST in mice (karim et al., 2018 ▶). Based on the previous reports, polyphenols may be the reason for antidepressant effects of the present herbal formula.

While the last studies indicated the anxiolytic effects of some of the ingredients of the present herbal formula (Becker et al., 2014 ▶; Ghazizadeh et al., 2020 ▶; Hattesohl et al., 2008 ▶; Komaki et al., 2015 ▶), it did not exert the significant anxiolytic-like behavior in the EPM. This conflict of results may be related to the type of extract solvent, the extraction methods, and the part of the herb used in the formulations which impact the structure, activity, and content of phytochemical constituents and their metabolites (Sultana et al., 2009 ▶). Since EPM induces both anxiety and fear-related behavior, the emotional state of animal may change from anxiety to fear during the EPM test. Therefore, it is suggested that the efficacy of this herbal formula should be evaluated with another anxiety test 

CUMS increases the activation of the HPA-axis and the secretion of its related molecules, such as cortisol and corticosterone (Wei et al., 2017 ▶). Based on the present results, the serum cortisol level had a significant decrease in the CUMS compared to the control group. Accordingly, it is verified that the hyper-activation of the HPA-axis occurred in CUMS-mice. Although the herbal formula as well as fluoxetine decreased the cortisol level, this reduction was not significant compared to the vehicle group. As a result, it could be suggested that the HPA axis is not the target of this herbal formula against CUMS. Although the increase in plasma cortisol level was reported in stressed mice and rats in the acute and chronic model, the secretion of cortisol in the laboratory rodents is insufficient, and corticosterone is known as the major glucocorticoid in mice and rats (Touma and Palma, 2005 ▶). Accordingly, it would be better to detect the level of corticosterone instead of cortisol. Since the drug treatment was performed along with the CUMS exposure, the suppression effect of the drugs on HPA axis may not be able to overcome the hyperactivity effect of the stress. So, for more accurate evaluation of HPA activity, it may be better to measure the level of Adrenocorticotropic hormone (ACTH) in serum and corticotropin releasing hormone (CRH) in hypothalamus. On the other hand, the increase of the duration of herbal formula treatment could be recommended to exert stronger effect on the HPA axis activity.

 Moreover, bupropion (15 mg/kg) had a significant increasing effect on serum cortisol levels vs. the control group. Bupropion inhibits the transporters responsible for dopamine reuptake and increases the synaptic dopamine concentration (Foley et al., 2006 ▶). The CRH neurons in the paraventricular nucleus obtain inputs from dopaminergic fibers. When dopaminergic receptors are stimulated, the release of CRH and, consequently, glucocorticoid production are elevated (Eaton et al., 1996 ▶). As a result, it is concluded that the chronic administration of bupropion can raise the serum concentration of cortisol by inducing an increase in dopamine’s synaptic level.

ALT and creatinine are the indicators of liver and kidney function whose content increased in the blood through the liver and renal toxicity, respectively. No significant changes occurred in ALT or creatinine serum level following herbal formula treatment. Interestingly, both ALT and creatinine levels significantly decreased in the fluoxetine group vs. the vehicle. The ALT level in the fluoxetine group was even lower than that in the control group. Weight loss and the lowering of the muscle mass are the main factors that reduce the serum level of ALT and creatinine (Uslan et al., 2007 ▶; Xu et al., 2012 ▶) which also occurred following CUMS (Antoniuk et al., 2019 ▶). Therefore, the fluoxetine group may have lost weight and experienced lowered muscle mass due to CUMS. Since several physiological factors affect the serum level of biochemical parameters, measurement of the baseline level of parameters in each group before treatment and its comparison with the post-treatment level could be beneficial for a more detailed analysis. However, no hepatotoxic and nephrotoxic effects were observed following the sub-chronic administration of herbal formula.

Chronic stress elevates the level of oxidative stress markers (Che et al., 2015 ▶). SOD is one of the main antioxidant enzymes that inhibit the oxidative stress. It was documented that CUMS disturbs the anti-oxidative defense system. The SOD level (Zhang et al., 2009 ▶) and activity (Bhatt et al., 2014 ▶) were attenuated in the liver and brain of CUMS-mice, respectively. In agreement with the previous reports, our results show that SOD activity was suppressed in the brain and kidney of the animals in CUMS group vs. non-CUMS. Interestingly, the herbal formula significantly increased the SOD level in the brain in the bupropion vs. the vehicle group in CUMS mice. Thus, it can be concluded that the antidepressant effect of herbal formula against CUMS may be due to its anti-oxidative effect in the brain. 

Previous studies reported that some herbal drugs in the herbal formula exerted antioxidant activity through impact on the SOD activity and releasing. Drinking *Lavandula angustifolia* extract for 14 days by rats increased the SOD level in their isolated hepatocytes (Kozics et al., 2017 ▶). The chronic oral administration of the *Melissa officinalis* L. hydroalcoholic extract had positive effects on the antioxidant defense parameters such as malondialdehyde (MDA) level, the total antioxidant capacity, the activities of SOD and glutathione peroxidase (GPx) in the brain of mice exposed to chronic restraint stress (Ghazizadeh et al., 2020 ▶). Moreover, several reports indicated the antioxidant activity of phytochemicals in herbal formula. Administration of gallic acid, and acetylsalicylic acid, phytochemicals of *Salix aegyptiaca*, significantly inhibited the MDA formation and increased the hepatic antioxidant enzymes such as SOD (Nauman et al., 2018 ▶). Polysaccharides isolated from the bark of *Cinnamomum zeylanicum* inhibited the DPPH radicals dose-dependently (Ghosh et al., 2015 ▶). Therefore, the stimulatory effects of the herbal formula on the brain SOD activity are consistent with the results of previous studies, while they are contrary to the previous results on the kidney and liver. It is worth mentioning that the antioxidant activity of the herbal extract is attributed to several factors such as the type and amount of phytochemicals, the type of solvent as well as the harvest time. The highest antioxidant capacity was observed in water and methanol extract of *Echium amoenum *and* Valerian officinalis,* respectively (Pilerood and Prakash, 2014 ▶; Suntar et al., 2018 ▶). The harvest time impacts the phytochemical content and antioxidant capacity of *Citrus aurantium* (Tang et al., 2021 ▶). In addition, the various components of *Citrus aurantium* like flavonoids, alkaloids, polysaccharides, coumarins, and neroli scavenge the different free radicals (Shen et al., 2017 ▶). In this context, the use of the different antioxidant tests is suggested.

According to the presented results, it is concluded that the sub-chronic administration of the Persian herbal formula causes antidepressant and anti-oxidative effects in mice under CUMS with no functional and histological changes in the liver or kidney.

## Conflicts of interest

The authors have declared that there is no conflict of interest.
